# Regulation of Cytokines in Cancer Pain

**DOI:** 10.1155/2016/2741205

**Published:** 2016-03-20

**Authors:** Xue-Jun Song, Robert H. LaMotte, Zhong Xie

**Affiliations:** ^1^Center for Anesthesiology and Pain Medicine, Cancer Hospital & Institute, Peking University, Beijing 100142, China; ^2^Neuroscience Research Institute, Peking University, Beijing 100191, China; ^3^Department of Anesthesiology, Yale University, New Haven, CT 06520, USA; ^4^Department of Physiology, Northwestern University, Chicago, IL 60611, USA

Cancer pain possesses a major challenge in clinical treatment, yet its underlying mechanisms remain elusive. Recent advances in understanding the molecular pathways mediating the inflammatory processes accompanied with cancer and cancer pain suggest the importance of proinflammatory cytokines. Further studies in this area will facilitate the discovery and validation of potential therapeutic targets for cancer pain. In this special issue, we present original research articles and clinical studies, as well as review articles, on the role of various cytokines and their regulation in the development and maintenance of cancer pain.

This special issue features two review articles. In the paper entitled “Treatment of Cancer Pain by Targeting Cytokines,” I. Vendrell et al. presented a comprehensive overview of the literature on the importance of cytokines in cancer pain and discussed the existing strategies to control the release of cytokines that have an impact on cancer pain. C. Panis and W. R. Pavanelli in “Cytokines as Mediators of Pain-Related Process in Breast Cancer” analyzed the major proinflammatory cytokines produced in breast cancer and discussed the evidences from current research regarding their role in the generation of pain-related clinical features.

The research article by G. Zhu et al. entitled “Radiotherapy Suppresses Bone Cancer Pain through Inhibiting Activation of cAMP Signaling in Rat Dorsal Root Ganglion and Spinal Cord” investigated the mechanisms of a major clinical treatment for bone cancer pain. They reported that, in a rat tumor cell implantation model, X-ray radiation reduced IL-1*β* and TNF-*α* concentrations and suppressed bone cancer pain by inhibiting the activation of cAMP-PKA signaling pathway in DRG and the spinal cord.

In the paper “Levo-Tetrahydropalmatine Attenuates Bone Cancer Pain by Inhibiting Microglial Cells Activation,” M. Zhang et al. examined the analgesic roles of L-THP in rats with bone cancer pain. This study described a possible clinical utility of L-THP administration in the treatment of bone cancer pain. The analgesic effects of L-THP on cancer pain may result from the inhibition of microglial cells activation and proinflammatory cytokines (TNF-*α* and IL-18) production.

S. Zhu et al. described the potential role of the spinal sigma-1 receptor in the development of bone cancer pain in a research article titled “Sigma-1 Receptor Antagonist BD1047 Reduces Mechanical Allodynia in a Rat Model of Bone Cancer Pain through the Inhibition of Spinal NR1 Phosphorylation and Microglia Activation.” The authors reported that intrathecal injection of sigma-1 receptor antagonist BD1047 attenuated mechanical allodynia and activation of microglial cells and suggested that targeting sigma-1 receptor may be a new strategy for the treatment of cancer pain.

The research paper by R. Pan et al., “Inducible Lentivirus-Mediated siRNA against TLR4 Reduces Nociception in a Rat Model of Bone Cancer Pain,” explored the potential of targeting TLR4 as a long-term treatment for bone cancer pain. The authors presented results showing the antinociception effect of a tetracycline inducible lentivirus carrying siRNA targeting TLR4. Proinflammatory cytokines as TNF-*α* and IL-1*β* in spinal cord were also decreased.

In the clinical study titled “Efficacy and Safety of Ropivacaine Addition to Intrathecal Morphine for Pain Management in Intractable Cancer,” Y. Huang et al. compared the efficacy and safety of an intrathecal continuous infusion of morphine and ropivacaine with intrathecal morphine alone in cancer patients and showed that combined morphine and ropivacaine administration through intrathecal access ports is efficacious and safe and significantly improved patients' quality of life.

In another clinical study titled “A Traditional Chinese Medicine Xiao-Ai-Tong Suppresses Pain through Modulation of Cytokines and Prevents Adverse Reactions of Morphine Treatment in Bone Cancer Pain Patients,” Y. Cong et al. reported that oral administration of Xiao-Ai-Tong alone or together with morphine can effectively suppress pain and reduce adverse reactions following morphine treatment. The study also found that Xiao-Ai-Tong treatment increased the anti-inflammatory cytokine interleukin-10 and decreased the proinflammatory cytokines interleukin-1*β* and tumor necrosis factor-*α* in blood.

Together, the reviews, research articles, and clinical studies featured in this special issue expand our knowledge and understanding of the mechanisms of cancer pain, especially the roles of the proinflammatory cytokines. We believe these data may contribute to improved management and treatment of cancer pain.

*Xue-Jun Song* *Xue-Jun Song* *Robert H. LaMotte* *Robert H. LaMotte* *Zhong Xie* *Zhong Xie*

